# Complete mitochondrial genome sequence of Black Bengal goat (*Capra hircus*)

**DOI:** 10.1080/23802359.2019.1623098

**Published:** 2019-07-10

**Authors:** Amam Siddiki, Masum Billah, Mohammad Alam, Kazi Shefaul Mulk Shawrob, Mahadia Kumkum, Sourav Saha, Muntaha Chowdhury, Atif H. Rahman, Michael Stear, Mohammad K. I. Khan, Gous Miah, AK M.Mollah, Abdul Baten

**Affiliations:** a Genomics Research Group, Faculty of Veterinary Medicine, Chattogram Veterinary and Animal Sciences University (CVASU), Chattogram, Bangladesh;; b Department of Biological Sciences, Asian University for Women (AUW), Chattogram, Bangladesh;; c Department of Computer Science and Engineering, Bangladesh University of Engineering and Technology (BUET), Dhaka, Bangladesh;; d AgriBio, Department of Animal, Plant and Soil Sciences, School of Life Sciences, La Trobe University, Bundoora, Australia;; e Department of Genetics and Animal Breeding, Faculty of Veterinary Medicine, Chattogram Veterinary and Animal Sciences University (CVASU), Chattogram, Bangladesh;; f AgResearch, Palmerston North, New Zealand;; g Southern Cross Plant Science, Southern Cross University, Lismore, Australia

**Keywords:** Black Bengal goat, *Capra hircus*, mitochondrial genome, protein-coding gene, tRNA, rRNA

## Abstract

The Black Bengal goat (*Capra hircus),* is a native breed found in Bangladesh, popular due to its economic contribution. Here, we report the complete mitochondrial genome sequence of Black Bengal goat. The circular genome is 16,640 bp long, comprising of 60.89% AT content. The genome contains 37 genes, consisting of 13 protein-coding genes, 22 tRNA genes, two rRNA genes, and a control region (D-loop).

## Introduction

The Black Bengal goat (BBG) is a ruminant, herbivore goat breed indigenous to northeastern India, West Bengal, Bihar, and Orissa and especially in Bangladesh. Its popularity in Bangladesh is due to high-quality meat, skin, milk, disease resistance capabilities and high prolificacy rate (Faruque and Khandoker 2007). Phenotypically they are dwarf, so its demand for food is low, and it takes less space than the other livestock (Hasan et al. 2014). They serve as an asset and play a vital role in the livelihoods of rural farmers in Bangladesh (Hassan et al. 1970). Owing to its unique traits with economic interest, we have investigated the mitochondrial genome information to comprehend its origin, molecular evolution, discerning phylogeny as well as physiology such as energy metabolism.

Samples were obtained from Research and Farm-Based Campus of CVASU, Hatazari, Chattogram, Bangladesh (22°30′28.9″N 91°46′58.5″E). Healthy BBG without known genetic diseases were incorporated for blood sampling. The collected blood specimen was preserved in EDTA tube and stored at −80 °C until used to isolate genomic DNA. Purified DNA was sent for library preparation and sequencing. DNA was sequenced using Illumina HiSeq 2500 by BGI Group Shenzhen, Guangdong, China. The organelle assembler NOVOPlasty V.2.7.2 was used to assemble the clean reads. As recommended by NOVOplasty developer, we kept the default kmer size of 49 (Dierckxsens et al. 2016). Web servers (MITOS) (Bernt et al. 2013), DOGMA (Wyman et al. 2004), and GeSeq (Tillich et al. 2017) were applied to perform structural and functional annotation. Subsequently, the annotated genes were then ensured through homology searches on GenBank and manual curation. Finally, mtDNA sequences were aligned and a phylogenetic tree was constructed using the program ClustalW implemented in MEGA V.10.0.5.

**Figure 1.**
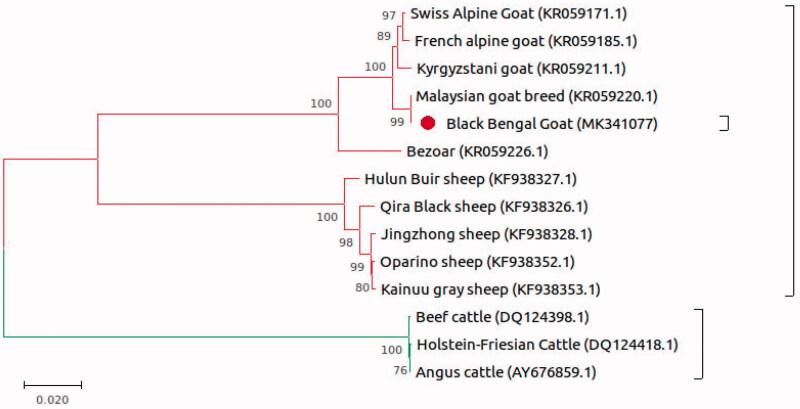
Phylogenetic analysis of Black Bengal goat based on the comparison of complete mitochondrial DNA sequence of 13 species. GenBank accession numbers were also mentioned.

The complete mitogenome of BBG (MK341077) is 16,640 bp in length and consists of 13 protein-coding genes, two ribosomal RNA genes (rRNA), 22 transfer RNA (tRNA) genes, and a control region (D-loop). The mitochondrial genome of BBG contains an A + T bias with an overall nucleotide composition of A = 5586 (33.57%), T = 4546 ( 27.32%), C = 4330 (26.02%), and G = 2178 (13.09%). The A + T-content of the mitogenome is 60.89%. Furthermore, the AT-skew is positive which is 0.1026 and GC-skew is negative which is −0.330. The majority of the protein-coding genes (PCGs) have been encoded on the H-strand of mtDNA. Only one PCG (*nad6*) and 7 transfer RNA genes (*trnA, trnC, trnE, trnN, trnP, trnS2,* and *trnY*) were encoded in the L-strand of mtDNA. Majority of the gene start with a typical start codon ATG while *nad2, nad3, nad5,* and *nad6* start with codon ATA. The 12S rRNA and 16S rRNA genes were, respectively, 954 bp and 1566 bp. The tRNA genes encoded in the genome ranged from 60 to 75 bp. The control region is between *trnaP* and *tnaF* and has a size of 1212 bp. BBG has a closer genetic relationship with the Malaysian goat breed and a further genetic distance to Swiss Alpine goat, according to phylogenetic tree analysis (Figure 1).

To sum up, this study provides the information of BBG mitogenome, which will also be important to do further taxonomic classification, phylogenetic reconstruction and implementing conservation strategies.
